# Transgenic expression of lactoferrin imparts enhanced resistance to head blight of wheat caused by *Fusarium graminearum*

**DOI:** 10.1186/1471-2229-12-33

**Published:** 2012-03-09

**Authors:** Jigang Han, Dilip K Lakshman, Leny C Galvez, Sharmila Mitra, Peter Stephen Baenziger, Amitava Mitra

**Affiliations:** 1 Department of Plant Pathology, University of Nebraska Lincoln, Lincoln, NE 68583, USA; 2 USDA-ARS, Sustainable Agricultural Systems Laboratory, Beltsville, MD 20705, USA; 3 Department of Agronomy & Horticulture, University of Nebraska Lincoln, Lincoln, NE 68583, USA

## Abstract

**Background:**

The development of plant gene transfer systems has allowed for the introgression of alien genes into plant genomes for novel disease control strategies, thus providing a mechanism for broadening the genetic resources available to plant breeders. Using the tools of plant genetic engineering, a broad-spectrum antimicrobial gene was tested for resistance against head blight caused by *Fusarium graminearum *Schwabe, a devastating disease of wheat (*Triticum aestivum *L.) and barley (*Hordeum vulgare *L.) that reduces both grain yield and quality.

**Results:**

A construct containing a bovine lactoferrin cDNA was used to transform wheat using an *Agrobacterium*-mediated DNA transfer system to express this antimicrobial protein in transgenic wheat. Transformants were analyzed by Northern and Western blots to determine lactoferrin gene expression levels and were inoculated with the head blight disease fungus *F*. *graminearum*. Transgenic wheat showed a significant reduction of disease incidence caused by *F. graminearum *compared to control wheat plants. The level of resistance in the highly susceptible wheat cultivar Bobwhite was significantly higher in transgenic plants compared to control Bobwhite and two untransformed commercial wheat cultivars, susceptible Wheaton and tolerant ND 2710. Quantification of the expressed lactoferrin protein by ELISA in transgenic wheat indicated a positive correlation between the lactoferrin gene expression levels and the levels of disease resistance.

**Conclusions:**

Introgression of the lactoferrin gene into elite commercial wheat, barley and other susceptible cereals may enhance resistance to *F. graminearum*.

## Background

Fusarium head blight (FHB) or head scab is one of the most devastating plant diseases of wheat (*Triticum aestivum *L.) and barley (*Hordeum vulgare *L.). Wheat holds a leading position in human nutrition in the world. Therefore, sustained wheat production is vital to ensure world food security. The International Maize and Wheat Improvement Center (CIMMYT) has identified FHB as a major factor limiting wheat production in many parts of the world [1]. Symptoms of the disease include brown colored lesions on wheat and barley spikelets. The disease is caused by the fungus *Fusarium graminearum *inflicting tremendous economic losses by reducing grain yield and quality in these two important crops. The fungus readily forms its sexual stage (*Gibberella zeae*) producing ascospores that are forcibly shot into the air with the increased ability to disseminate from colonized residue where perithecia form. Losses are compounded by mycotoxins (e.g. deoxynivalenol, DON) that are produced by the fungus in diseased grains. In general, there is a 1 ppm limit for DON in all finished wheat products that may be consumed by humans. Head blight causes severe and increasing crop losses worldwide [2]. Since 1990, wheat and barley farmers in the United States alone have lost over $3 billion dollars due to FHB epidemics. The disease has recently reemerged in the Midwestern and Eastern states of the USA and continues to cause extensive losses [3,4].

There are no reports of true resistance against *F. graminearum *within cultivable species and there are only very few commercial agronomic cultivars partially resistant to the pathogen. There are only a few fungicides, Prosaro^® ^(Prothioconazole + Tebuconazole) & Proline^® ^(Prothioconazole) from Bayer and Caramba^® ^(Metconazole) from BASF, reported to be effective against FHB and no suitable alternatives exist to control the pathogen in organic production systems. Even the optimal fungicide applications may only provide a 50-60% reduction in FHB incidence [5]. Use of biological control has often proved inconsistent or did not work at all under field conditions [6]. Although none of the available commercial cultivars is immune to infection, different varietal reactions to FHB are known. Two main types of resistance, Type I and Type II, are most commonly recognized [7-9]. Type I resistance reduces the number of initial infections as measured by the number of infected spikelets following a spray inoculation. Type II resistance restricts spread of the fungus in infected tissue and is measured by the number of spikelets infected in a spike beyond an initial inoculated infection site on the spike. Other types of resistance or tolerance are also known to occur based on the ability to resist kernel infection, degrade mycotoxins or to maintain a decent yield despite FHB infection [7].

In the absence of adequate natural genetic resistance, transgenic introduction of resistance may be a sustainable alternative to chemical approaches to disease management [10]. There are a number of reports describing transgenically induced resistance with various genes against several fungal diseases emphasizing the importance of testing transgenic resistance against FHB [11-17]. Recently published approaches such as, expression of a pectin methyl-esterase [18], a polygacturonase inhibiting proteins [19], an antifungal radish defensin [20], a truncated form of yeast ribosomal protein L3 [21] and a phytoalexin Zealexin [22] have all shown to provide quantitative resistance against FHB. Makandar et al. [23] showed that over-expression of the Arabidopsis NPR1 gene in wheat, a master regulator of systemic acquired resistance, confers resistance to FHB. Detoxification of DON in genetically engineered crops [24,25] has also been shown to be effective against FHB. A recent review by Kazan et al depicted current advancements in FHB pathogenomics and host resistance [26].

Numerous experiments have shown the benefits of antimicrobial peptides for disease resistance [27]. Introduction of resistance against a broad range of plant pathogens is also encouraged for disease management. Lactoferrin, a cationic iron-binding glycoprotein of 80 kDa belonging to the transferrin family [28], is present in milk, tears, saliva, and mucous secretions of most mammals and plays a major role in the immune system of newborns by modulating immune functions. The N-terminal peptide of Lactoferrin, which can be released by proteolytic cleavage, is highly bactericidal [29]. This peptide, lactoferricin, is the shortest active amino acid sequence that is resistant to further enzymatic cleavage [30]. Another prominent property of lactoferrin or lactoferricin is its potent activity against a wide range of microorganisms including both gram-negative and gram-positive bacteria, as well as fungi and viruses [31]. In addition to its anti-microbial as well as anti-inflammatory properties, Lactoferrin may have role in iron absorption and/or excretion and in gastric health of newborns [32]. Recombinant lactoferrin has been produced in filamentous fungi, plants and animals for biopharmaceutical purposes [33-35]. We have previously shown that lactoferrin expressed in tobacco inhibited several phytopathogenic bacteria in vitro [35]. In addition, transgenic tobacco and tomato plants expressing lactoferrin significantly delayed wilt symptom development caused by *Ralstonia solanacearum*, in a dose-dependent manner [36,37]. Similarly, expressed lactoferrin in transgenic pear showed an increase in resistance against *Erwinia amylovora *[38]. Takase et al [39] evaluated transgenically expressed human lactoferrin and lactoferricin in rice against disease-causing *Burkholderia plantarii *(causal agent of Bacterial Seedling Blight), Rice dwarf virus and *Pyricularia oryzae *(*Magnaporthe oryzae*, causal agent of Rice Blast). However, they found significant resistance only against *B. plantarii*. Bovine lactoferricin-derived peptides were shown to have significant *in vitro *antimicrobial activity against many plant pathogenic filamentous fungi including *Fusarium *and *Magnaporthe *species [40]. In the present investigation, we evaluated if a bovine lactoferrin gene can be utilized for controlling FHB caused by the fungus *F. graminearum*. We developed and tested transgenic wheat to evaluate possible resistance conferred by expressed lactoferrin against *F. graminearum in vitro *and *in planta*. The results demonstrated that lactoferrin imparts partial resistance to wheat against the FHB pathogen.

## Results

### Transformation and regeneration of transgenic wheat

**Figure 1:**
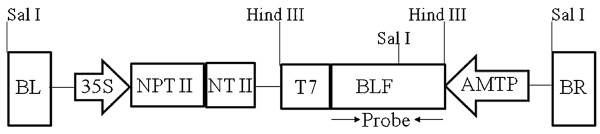
**Schematic diagram of the Transfer-DNA segment of the binary plasmid pAM4424**. The plasmid contains an antibiotic resistance gene neomycin phosphotransferase (*NPT *II) for the selection of wheat transformants and an antimicrobial bovine lactoferrin (*BLF*) gene for resistance against *F. graminearum*. BL & BR, T-DNA left and right borders; 35S, Cauliflower Mosaic Virus 35S promoter; NT & T7, T-DNA genes nopaline synthase and Transcript 7 terminators; AMTP, *Chlorella *virus Adenine Methyltransferase gene promoter. Probe: DNA sequence used for Northern blot.

The plasmid vector pAM4424 containing a bovine lactoferrin gene and an nptII selectable marker gene (Figure 1) was transformed into Bobwhite wheat cultivar by the *Agrobacterium*-mediated transformation method [31]. A total of 117 independent transformants were generated over a period of two years. To insure independent events, only one transformant was retained from each immature embryo used for transformation. The transgenic wheat seedlings grew normally to maturity and produced fertile seeds, except 8 lines (6.8%) that failed to produce viable seeds.

All transgenic lines were screened using southern blot and fungal inoculation to determine the number of T-DNA inserts and for resistance against FHB. Of the 117 transgenic lines, 33 contained a single copy transgene and were selected for further testing. These transgenic lines were selfed to obtain homozygous lines. Seven single-copy independent transgenic lines with highest levels of FHB resistance were selected for further studies.

### Expression of Lactoferrin in transgenic plants

**Figure 2:**
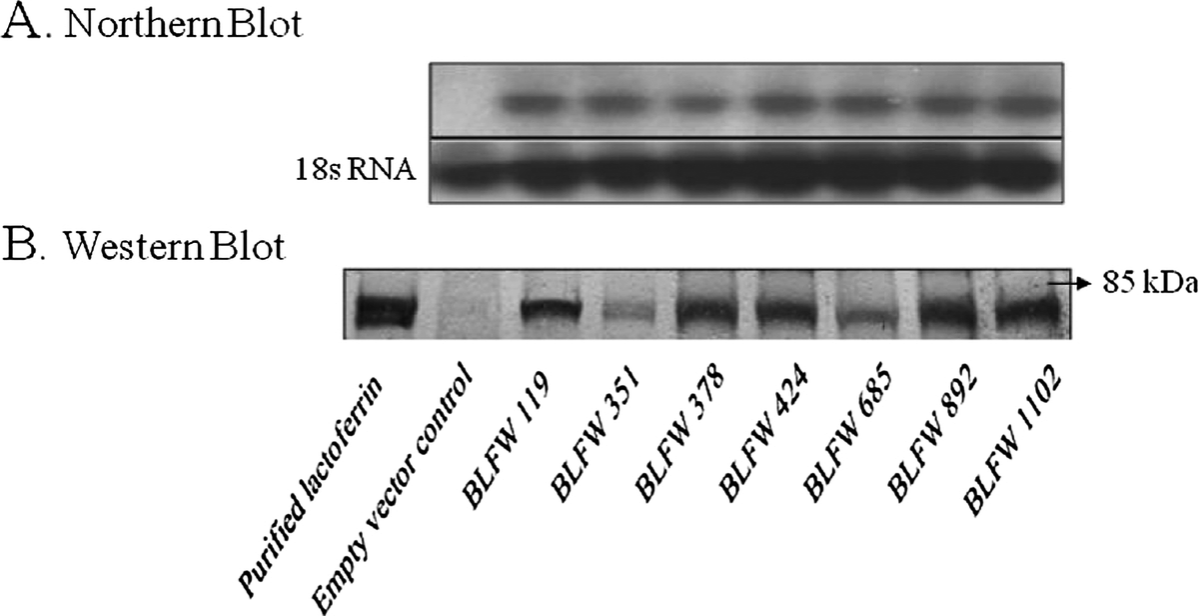
**Expression of lactoferrin in seven T_8 _transgenic wheat lines determined by Northern blot (A) and Western blot (B) analyses using two top leaves and inflorescences at growth stage Feekes 10.5**. Northern blot: Total RNA from transgenic and control plants were hybridized with a ^32^P-labeled lactoferrin cDNA probe. 18s RNA was used as a loading control. Western blot: Immunodetection of lactoferrin protein in transgenic wheat plants using a polyclonal antibody reagent. Lane PC, purified lactoferrin protein; lane C, control wheat plant, lane 1-7 transgenic wheat lines: BLFW 119, 351, 378, 424, 685, 892 and 1102. Position of 85 kDa molecular weight marker is shown with an arrow on the right.

The T_8 _progeny of the 7 selected transgenic wheat lines were subjected to molecular analyses to confirm and compare expression of lactoferrin protein. Northern blot analysis was conducted to investigate the expression pattern of the lactoferrin gene using a gene fragment to generate a gene-specific probe. The result indicated that lactoferrin was expressed in all seven transgenic lines. There was only minor variation in the lactoferrin mRNA levels among these lines (Figure 2A) presumably due to stable expression of a single copy transgene in T_8 _generation.

Results of the immuno-blotting experiment with lactoferrin-specific antibody demonstrated that lactoferrin is expressed in all transgenic wheat lines tested. Transgenic wheat expressing lactoferrin protein demonstrated the presence of a band at 80 kDa, the predicted molecular weight of lactoferrin (Figure 2B). The bands co-migrated with a band from purified lactoferrin protein. Transgenic lines produced lactoferrin ranging from 21 to 67 ng per mg of leaf tissue. The line BLFW 1102 showed the highest level whereas the line BLFW 351 had the lowest level of lactoferrin protein. Although the steady state lactoferrin mRNA levels in these lines did not differ significantly, the protein levels varied 3 fold.

### In vitro agar-gel diffusion inhibition assay

**Figure 3:**
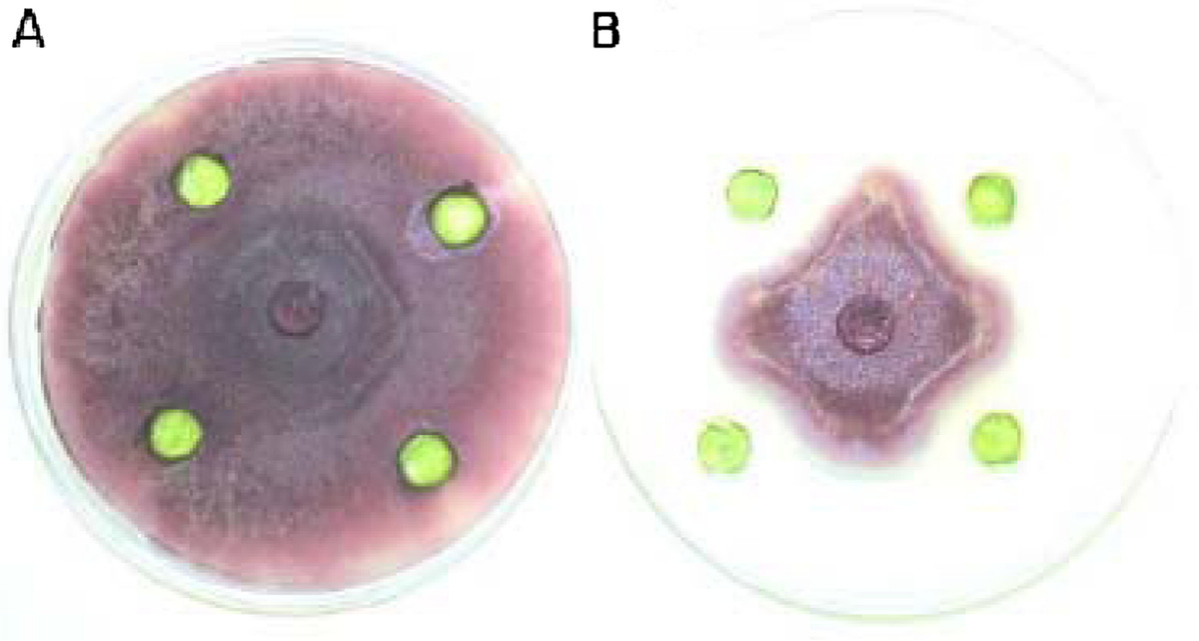
**Antifungal activities of soluble protein extracts from transgenic wheat leaves expressing the lactoferrin gene**. One hundred μg of total protein from four controls (**A**) and four lactoferrin-transgenic wheat (**B**) was used in each well. The plates show significant inhibitory effect of transgenic protein extracts on the growth of *F. graminearum *in vitro.

Fungicidal activity of total protein extracts from the transgenic wheat leaves was determined *in vitro *using an agar-gel diffusion inhibition assay. In Figure 3 the wells in plate-A contained total protein extracts from four control wheat plants, and plate-B contained extracts from four transgenic wheat lines expressing lactoferrin. The inhibitory effect of transgenic leaf extracts expressing lactoferrin was clearly noticeable in plate-B. Inhibition was not detected in the presence of either control plant extracts not expressing lactoferrin protein or protein extraction buffer used to prepare the extracts (not shown). The assay showed significant reduction of fungal growth in the presence of lactoferrin protein extracts from transgenic wheat plants. The fungal growth reached near the wells containing the extracts, but failed to advance further. Efforts to retrieve viable fungal cultures using peripheral hyphal tips from plate-B were unsuccessful after 8 days of growth in plate-B, presumably due to the fungicidal activity of lactoferrin.

### Greenhouse evaluation of transgenic wheat expressing lactoferrin

**Figure 4:**
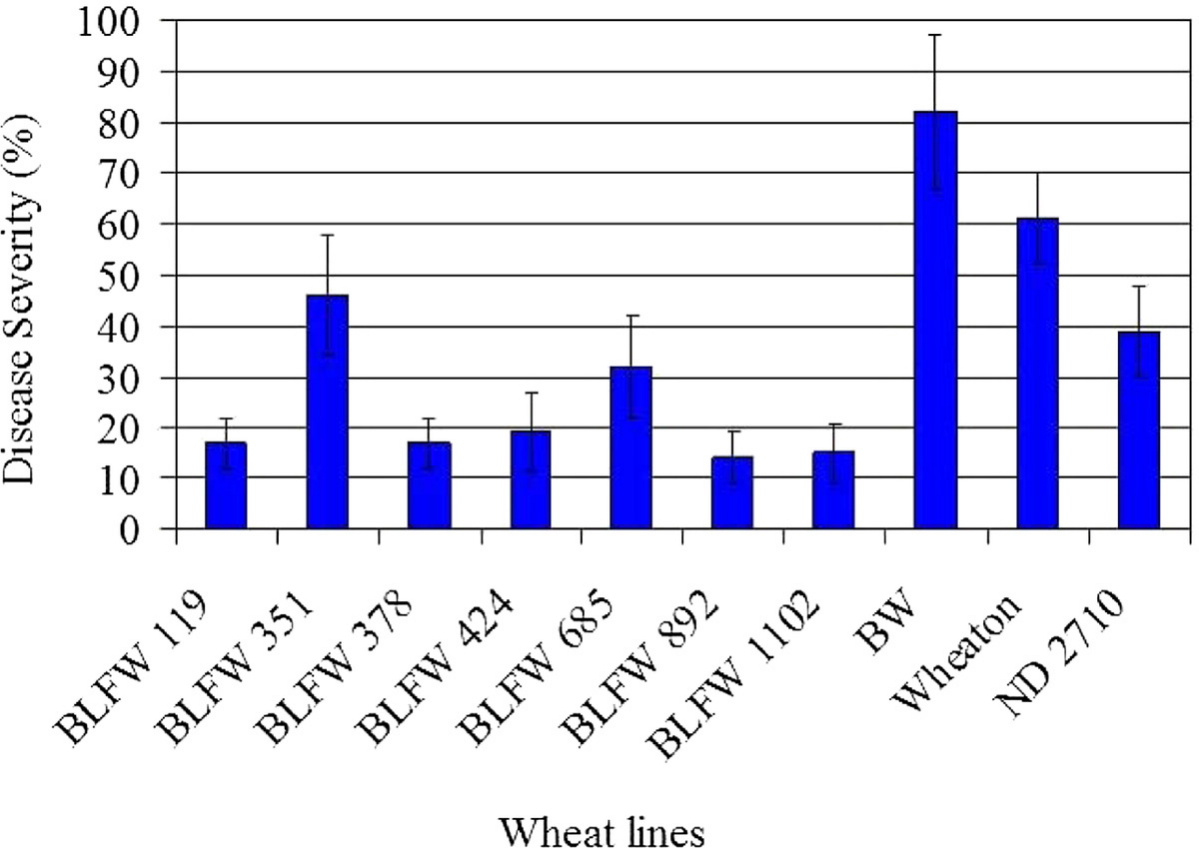
**Disease severities in seven transgenic wheat lines**. Transgenic lines and three control wheat varieties were spray-inoculated with a conidial suspension of *F. graminearum*. Disease severity was calculated as percent of infection in the sprayed heads. Six of the seven transgenic lines showed significant FHB resistance compared to two commercial wheat varieties, Wheaton and ND 2710, and a transgenic Bobwhite control carrying an empty vector. BLFW, transgenic wheat lines; BW, wheat cultivar Bobwhite carrying an empty vector; Wheaton and ND2710, susceptible and tolerant wheat breeding lines, respectively. Error bars represent standard errors of mean. All BLFW lines are significantly different from BW at P < 0.01 by Student's t-tests.

A large number of transgenic wheat plants were generated and subjected to successive screenings and selection of FHB resistant plants starting with the primary transformants for a period of over four years. Only plants that showed resistance of at least 50% compared to Bobwhite control were carried forward by selfing to the next generation. The plants showing the highest levels of resistance were then screened for the copy number of the transgene. Seven lines with a single copy transgene and highest resistance levels were further selected and selfed until they became homozygous. These seven homozygous transgenic wheat lines of T_8 _generation were tested for scab resistance in greenhouse conditions. The greenhouse testing was done in eight independent experiments using over 400 transgenic wheat plants. The disease severity was determined as the percentage of infected spikelets per head. All visually detectable discolored spikelets were counted as infected spikelets. A typical resistant reaction from a single experiment is shown in Figure 4 using 4 replicates of each transgenic line. A transgenic Bobwhite wheat line containing an empty vector was used as a control along with the untransformed Wheaton and ND 2710 cultivars. Both Wheaton and ND 2710 are conventional hard red spring wheat varieties. While Wheaton is susceptible to FHB, ND 2710 was bred for FHB resistance at the North Dakota State University [41,42].

Mean percent of infection in transgenic lactoferrin wheat varied from 14 - 46% while mean percent infections in Bobwhite, Wheaton and ND 2710 were 82%, 61% and 39% respectively. The level of resistance in individual lines was consistent in all experiments within the error margin. Disease resistance in transgenic lines was also consistent in several independent greenhouse assays at various times of the year. These lines not only consistently showed significant resistance compared to the cultivar Bobwhite, six out of seven lines also provided higher levels of resistance than Wheaton and ND 2710. Furthermore, five transgenic lines, BLFW- 119, -378, -424, -892, and - 1102, were about 3 times more resistant than ND 2710. Most discoloured (bleached) spikelets produced normal grains. Occasionally the grains were slightly smaller but never shrivelled and there was no visible fungal growth on the grains. A few discoloured spikelets did not produce any grains. More importantly, DON levels in five transgenic lines, BLFW- 119, -378, -424, -892 and - 1102, were below the 1 ppm limit established by the Food and Drug Administration (FDA, USA) for finished wheat grain products for human consumption. Under similar greenhouse conditions artificially spray-inoculated control BW lines had an average of 28.5 ppm DON. Although natural infection in Nebraska wheat fields varies widely, we routinely detect over 5 ppm DON in mildly infected wheat fields and over 50 ppm in moderately infected wheat fields.

### Differential expression of lactoferrin in wheat leaves and glumes

**Figure 5:**
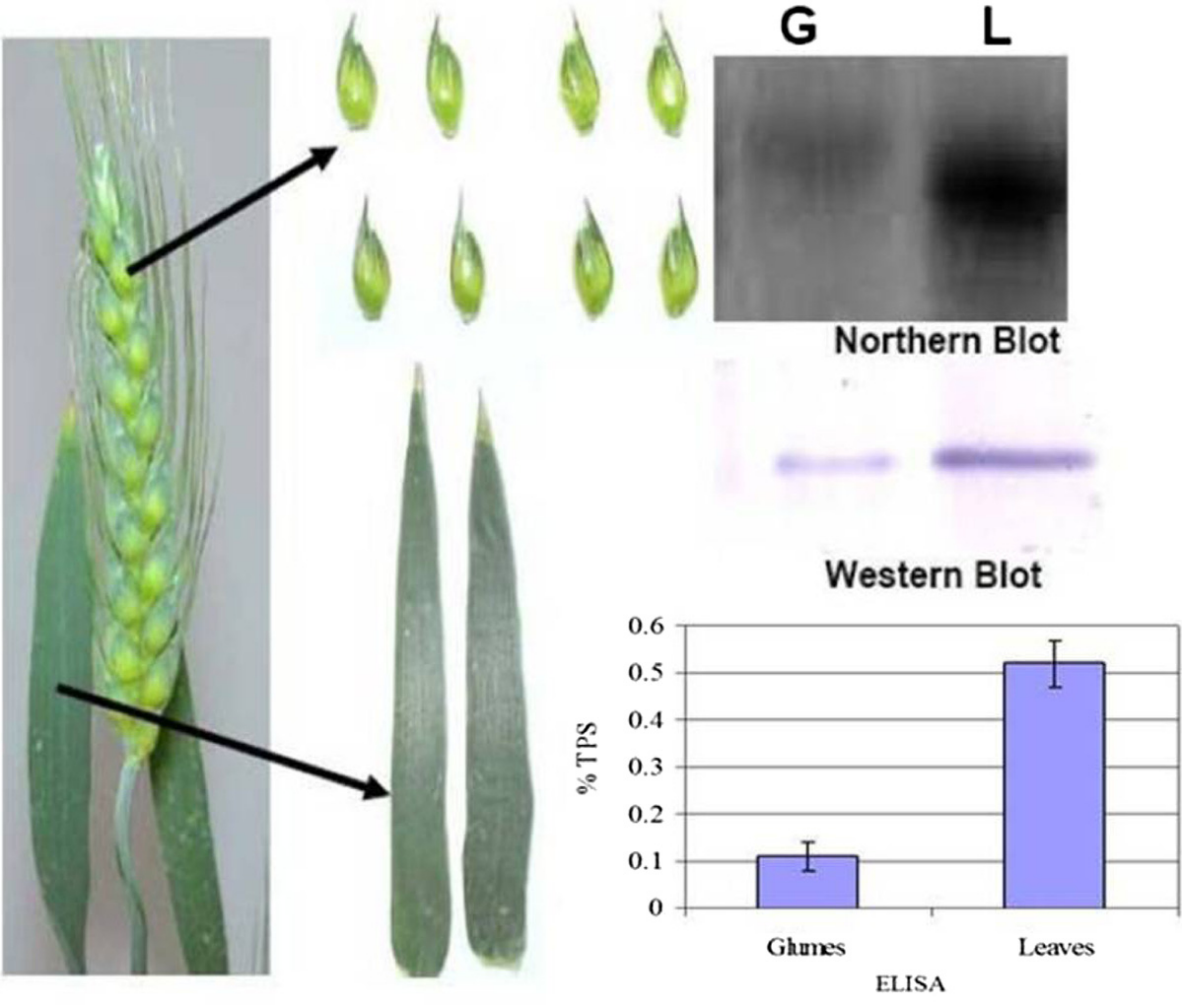
**Differential expression in wheat apical leaves and floret glumes**. Glumes (G) and leaves (L) from a single transgenic wheat plant (growth stage Feekes 10.5) were assayed for the level of lactoferrin expression by Northern and Western blot analyses. Quantitative estimation using ELISA showed that lactoferrin expression in glumes was only 20% of that in leaves.%TSP, Percent lactoferrin in total soluble protein.

As glumes are the outermost structures of wheat florets, they are exposed to the fungus spore at the onset of the disease pathogenesis. Hence, the transgene expression level must be adequate in the glumes to be effective in providing resistance against the scab fungus. We separated glumes from transgenic wheat florets and determined the level of lactoferrin expression at the protein and RNA levels by Western and Northern blots, respectively. Quantitative determination of lactoferrin protein in transgenic wheat leaves and glumes was made using ELISA. The level of lactoferrin expression in the glumes was significantly less than the expression in the leaves. Both Northern blot and Western blot assays clearly depicted this difference (Figure 5). The lactoferrin concentration in the glumes was 0.11% of total soluble protein, whereas lactoferrin concentration in the leaves was 0.52% of the total soluble protein. Hence, a much reduced level of lactoferrin protein is available in wheat glumes. Detection of low levels of lactoferrin in the glumes could be due to a lower level of the promoter activity in glumes. Alternatively, there may also be differential protein stability in the glumes or the overall soluble protein extraction from glumes is inefficient due to the presence of high amounts of phenolic compounds and lignin polymers.

**Figure 6:**
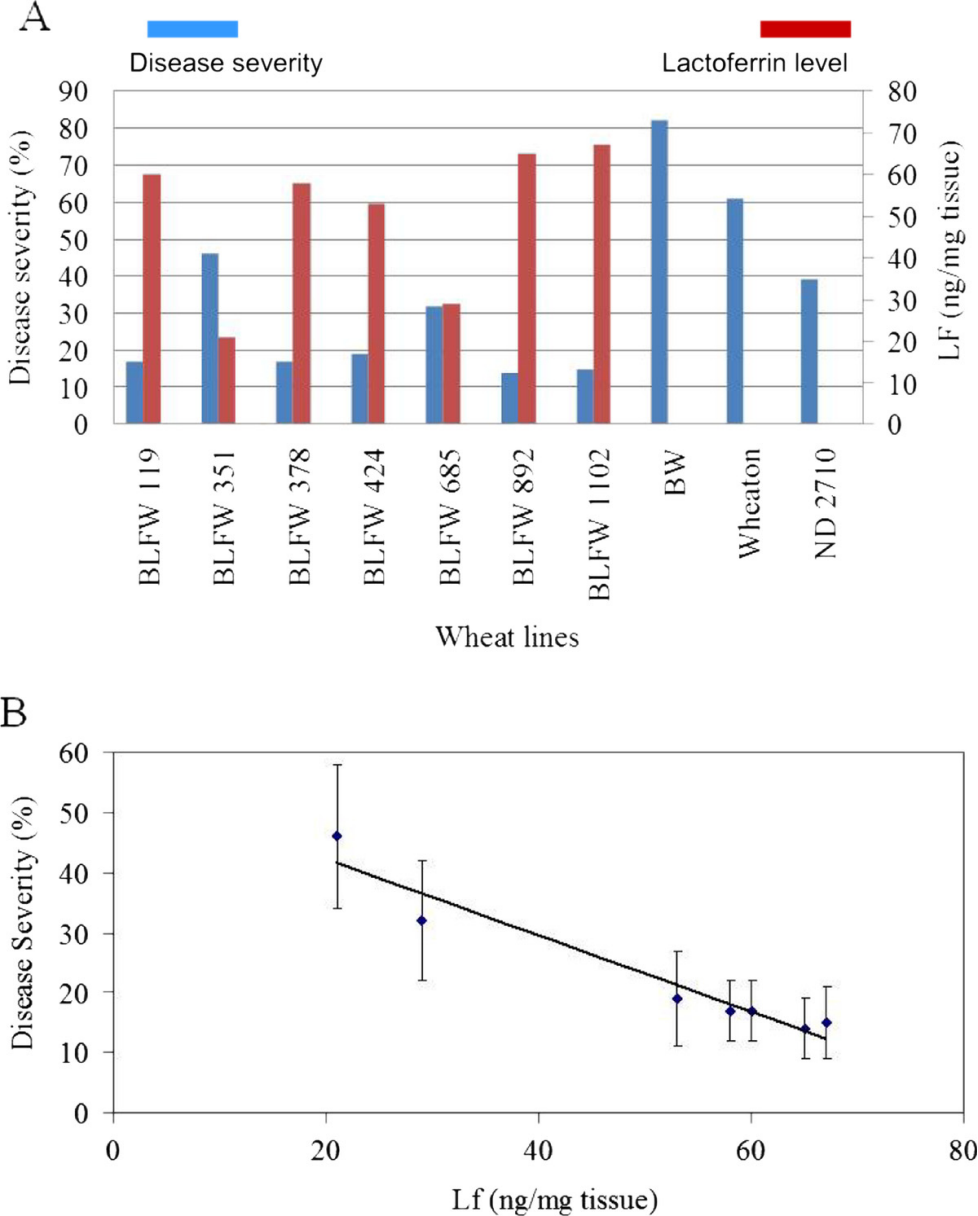
**Association of disease resistance with lactoferin levels**. **A**. The level of disease resistance (blue bars) is proportional to the level lactoferrin concentration (red bars) in the transgenic wheat lines. Lactoferrin concentration (LF) was determined in samples consisting of a flowering head and two top leaves (growth stage Feekes 10.5). **B**. Regression analysis showed a linear relationship between disease resistance and lactoferrin concentration.

The concentration of lactoferrin in the apical wheat consisting of the inflorescence and two top leaves (growth stage Feekes 10.5) were also determined and compared with the FHB severity (Figure 6A). The highest level of lactoferrin was found in the transgenic line BLFW 1102 (67 ng/mg tissue) and the lowest in the line BLFW 351 (21 ng/mg tissue). There was a clear correlation between the concentration of lactoferrin and the level of resistance in various transgenic lines, higher levels of lactoferrin protein, in general, resulted in higher levels of FHB resistance (Figure 6B).

## Discussion

Fusarium head blight has emerged as a major threat to wheat and barley crops around the world. The disease can occur on all small grain crops when the spore of the fungus germinates and infects developing kernels on the wheat head. FHB not only reduces grain yield and quality, but is frequently associated with fungal toxins which are hazardous to the health of humans and animals [43]. The United States Department of Agriculture (USDA) ranks FHB as the worst plant disease to hit the USA since the stem rust (*Puccinia graminis*) epidemics over fifty years ago [44]. Improving FHB resistance is a high priority in wheat and barley breeding programs. In this paper we used a broad-spectrum antimicrobial gene for FHB resistance in transgenic wheat. A bovine lactoferrin gene was introgressed into a wheat cultivar susceptible to FHB using *Agrobacterium *mediated transformation method. A large number of transgenic plants expressing the lactoferrin protein were generated. Constitutive expression of this antimicrobial protein did not visually alter morphology and physiology of wheat plants.

We demonstrated that lactoferrin inhibits the growth of a fungal plant pathogen *F*. *graminearum *both in vitro and in vivo. We also showed that transgenic expression of lactoferrin in a spring wheat cultivar Bobwhite imparts considerable resistance against FHB. All transgenic lines exhibited a significant level of resistance compared to untransformed Bobwhite and two cultivated wheat varieties Wheaton and ND 2710.

The total soluble protein from transgenic wheat leaf tissues was very effective in inhibiting the fungus in vitro; however, the transgenic plants were not immune to the disease. To investigate this difference further, we determined the actual level of lactoferrin in transgenic wheat leaves and in floret glumes. We had observed that the glumes are infected first during the manual inoculation of wheat inflorescence with a spore suspension. Hence, we tested the glumes of the transgenic spikelets for lactoferrin protein expression. The actual lactoferrin expression in wheat glumes was about one-fifth the expression level in leaves. It is anticipated that the resistance levels in the transgenic wheat lines can be significantly enhanced if the glumes had similar lactoferrin levels as the leaves. It is conceivable that using a glumes-specific promoter would further significantly enhance lactoferrin effectiveness against FHB in transgenic wheat. Huang [45] reported a five-fold higher level of lactoferrin in transgenic rice grains using a proprietary ExpressTec system.

The variation in the resistance levels among the independent transformation events was correlated with the actual amount of lactoferrin in the transgenic lines. The results of northern and western blots corroborated the resistance of transgenic wheat lines expressing bovine lactoferrin protein. The northern-blot analysis of the seven advanced lines did not show a significant variation in the levels of lactoferrin mRNA in the transgenic plants (Figure 2A). The actual accumulation of lactoferrin protein, however, varied among the seven transgenic lines as evident from the western blot assay (Figure 2B). As these lines were selected after many screenings and self-pollination cycles, the level of variation in these selected lines was relatively small. It is possible that transgenic lines BLFW-351 and - 685 produced mRNAs that were not translated efficiently due to mutations in the 5' untranslated region of lacoferrin mRNA. Overall, there was a good correlation between the levels of lactoferrin protein produced in transgenic wheat lines and the level of resistance against FHB. A Mendelian segregation pattern for a single locus insertion was observed in all seven transgenic lines selected for disease resistance studies and the trait was stable after the eighth generations. High level expression of foreign proteins in plants often leads to gene silencing [46], so expression stability must be assured in transgenic lines. Gene silencing was not observed in lactoferrin expression in the seven advanced wheat lines in the course of experimentation for eight generations.

Lactoferrin appears to be one of the promising non-plant antibacterial genes with the potential for the control of aggressive fungal pathogens such as *F. graminearum*. Experiments and results described here show promise for a new approach to manage a potentially devastating disease caused by *F. graminearum*. Use of a lactoferrin transgene may provide a critically important tool for the integrated management of FHB in sustainable production environments. Recent incorporation of a source of resistance from Sumai 3 into spring wheat cultivars such as Alsen, Faller, Glenn, Howard and Steele-ND has already improved FHB resistance in these varieties [47,48]. Introducing the lactoferrin gene into these varieties will likely enhance FHB resistance of these varieties.

Lactoferrin is ubiquitous. Copious amount of lactoferrin can be readily detected in milk and other routinely consumed dairy products [49]. The level of lactoferrin protein in transgenic wheat lines is substantially lower than lactoferrin in supermarket milk. Transgenic expression of lactoferrin also seems to impart a broad-based resistance against plant bacterial and fungal pathogens. This together with the fact that lactoferrin is one of the safest and occurs in the diet of humans; make this gene a potentially highly desirable candidate to introduce plant resistance against diseases.

## Conclusions

Wheat production in the United States has already become less profitable and unpopular due to the sporadic low market prices, increasingly stringent requirements for very low mycotoxin levels, and lack of effective and economically viable methods for controlling pathogens. The lactoferrin-expressing transgenic wheat provides wheat breeders an avenue to incorporate FHB resistance into commercial wheat cultivars combining biotechnology with conventional breeding approaches. As the northern US states primarily grow hard red and white winter wheat cultivars, crosses to elite winter wheat are necessary to understand how the lactoferrin gene works in those cultivars under winter field conditions.

## Methods

### Construction of vector and development of transgenic plants

A binary vector containing a bovine lactoferrin gene [35] was used for transformation of wheat. The lactoferrin gene was driven by the promoter of the adenine methyl transferase gene of *chlorella *virus PBCV1 [50] and a DNA sequence from *Agrobacterium *T-DNA gene 7 was used as a transcription termination signal creating a binary plasmid pAM4424 (Figure 1). The binary vector also contained a selectable marker gene *npt*II driven by the cauliflower mosaic virus 35S promoter. The plasmid was transferred to *Agrobacterium *strain C58C1 [51] and used for wheat transformation. Bobwhite, a FHB susceptible soft white spring wheat cultivar, was used to generate transgenic plants using an *Agrobacterium*-mediated transformation protocol [52]. Immature embryos, approximately 14 days post anthesis, were used as explants. The embryos were precultured on callus induction medium for four days followed by co-cultivation in freshly prepared *Agrobacterium *inoculum harbouring the binary plasmid pAM4424. Following co-cultivation the explants were processed and placed on selection medium containing 10 mg/L G418. Regenerating shoots were transferred to media in Magenta boxes for rooting and subsequently transferred to soil [52].

### Analysis of lactoferrin gene expression in transgenic wheat

Transgenic wheat plants were examined for the expression of the transgene by Northern blot assay. Subsequently, the presence of lactoferrin protein was also detected by Western blot. An empty vector (pAM4424 without the BLF gene) containing transgenic wheat line was used as a control.

#### Northern blot analysis

Leaf tissues (500 mg) and glumes (250 mg) from immature grains were collected from each transgenic plant and ground in a mortar with liquid nitrogen. Trizol reagent (Invitrogen, Carlsbad, CA) was used to isolate total RNA using the manufacturer's instructions. Fifteen μg of total RNA was separated on a formaldehyde agarose gel (1%) at 85 volts for 2 hours, the gel was pre-soaked in 20XSSC solution for 15 min followed by transfer to a Zeta-Probe GT membrane (Bio-Rad, Hercules, CA) using a TurboBlotter (Schleicher & Schuell, Inc., Keene, NH) and cross-linked by an UV crosslinker (Stratagene Stratalinker, La Jolla, CA). Gel eluted bovine lactoferrin fragment (2022 bp) (Figure 1) was used for the ^32^P-labeled probe. Hybridization was performed at 65C overnight and the membrane was washed three times each with the washing solution at 65C. The washed membranes were exposed to Kodak X-OMAT film in a cassette at -80C for 24 hours.

#### Western Blot assay and Enzyme-Linked Immunosorbent Assay (ELISA)

Total proteins from transgenic plants were extracted according to Mitra and Zhang [35]. Extracts containing 50 μg of total soluble proteins were separated on 12.5% (w/v) acrylamide gels [53] along with a 200 ng of commercially available lactoferrin (Sigma) as a standard. Blotting to membrane, immuno-hybridization and color development followed the protocol as described in Mitra and Zhang [35]. Transgenic wheat plants were screened to determine the levels of lactoferrin expression. Wheat flower heads with two top leaves (at growth stage Feekes 10.5) were used for quantitative lactoferrin concentration determinations.

Commercially available polyclonal antibodies (Sigma) were used following the manufacturer's instructions. A standard curve generated with purified lactoferrin protein was used to determine the lactoferrin concentration in transgenic wheat tissues.

### Bioassay for in vitro antifungal activity of total protein extracts from transgenic wheat leaves

#### Agar-gel diffusion assay

An agar-gel diffusion assay was set up to test in vitro tolerance of *F. graminearum *to lactoferrin expressed in transgenic wheat plants [54]. Total soluble protein extracted from transgenic and control wheat leaves were used in the assay. A 5 mm diameter agar plug of three-day old *F. graminearum *was placed in the middle, and protein extracts were added to the peripheral wells of a potato dextrose agar plate and incubated for 96 hours at 25C in the dark. There were three replicates of each protein extract and the whole experiment was repeated three more times.

### Inoculum preparation, inoculation and disease incidence assays of transgenic wheat

#### *F. graminearum *inoculum

Inoculum consisted of a conidial suspension made from ten isolates of *Fusarium graminearum *collected from Nebraska fields. Single spore culture was used to establish pure cultures of the ten isolates. A 5 mm diameter mycelial plug of each isolate was transferred onto 1/2-strength potato dextrose agar in 9 cm diameter Petri dishes and incubated at 25C for 10 days. Distilled water was added to each plate and the conidia were dislodged with a rubber-policeman. The suspension was filtered through one layer of cheesecloth and conidia concentration was adjusted to 7 × 10^4 ^conidia/ml.

#### Plant inoculation

Transgenic wheat lines were grown in 15 cm diameter round clay pots in a greenhouse. The experimental unit was one plant per 15 cm diameter pot. Treatments consisted of transgenic lactoferrin plants and controls and were arranged in a randomized complete block design with 4 replicates. Plants were maintained in a greenhouse room set at 26 ± 3°C and a 14-hour photoperiod. Each entire head was spray-inoculated at flowering with 2 ml of the conidia suspension using a hand-held spray bottle. Each inoculated head was sealed in a 16 × 9.5 cm^2 ^Ziploc bag for 72 hours.

#### Disease incidence

Disease severity was recorded 19 days after inoculation as a percentage of diseased spikelets on a single head. All discoloured spikelets were considered infected.

## Authors' contributions

JH, LCG, and SM carried out most of the experiments. DKL conducted ELISA for lactoferrin and DON and participated in manuscript preparation. PSB grew wheat plants and contributed to wheat plant analysis and manuscript preparation. AM conceived of the study, and participated in its design and coordination and participated in manuscript preparation. All authors have read and approved the final manuscript.
